# Emotional Stress During Pregnancy – Associations With Maternal Anxiety Disorders, Infant Cortisol Reactivity, and Mother–Child Interaction at Pre-school Age

**DOI:** 10.3389/fpsyg.2019.02179

**Published:** 2019-09-25

**Authors:** Anna-Lena Zietlow, Nora Nonnenmacher, Corinna Reck, Beate Ditzen, Mitho Müller

**Affiliations:** ^1^Institute of Medical Psychology, Center for Psychosocial Medicine, Heidelberg University Hospital, Heidelberg, Germany; ^2^General Psychiatry, Center for Psychosocial Medicine, Heidelberg University Hospital, Heidelberg, Germany; ^3^Department of Psychology, Ludwig Maximilian University of Munich, Munich, Germany

**Keywords:** stress, cortisol reactivity, child development, pregnancy, anxiety disorders

## Abstract

There is growing evidence that even milder forms of maternal stress or anxiety during pregnancy affect the fetus causing possible long-term consequences for infant and child development. The mechanisms through which prenatal maternal stress may affect the unborn are not yet entirely clarified. Due to limited self-regulatory skills after birth, infants depend on sensitive behavior of their parents to regulate affective states and physiological arousal. Dyadic affect regulation has been linked to various developmental patterns up to adolescence and thereby represents a key element of early social relationships. Aim of the study was to evaluate possible long-term consequences of emotional stress during pregnancy and postpartum anxiety disorders, as well as infant postpartum cortisol reactivity on mother–child-interaction at pre-school age. The sample comprised of *N* = 63 mother–infant dyads at study entry, *n* = 28 diagnosed with postpartum anxiety disorders according to the Diagnostic and Statistical Manual of Mental Disorders IV (DSM-IV), *n* = 35 were healthy controls. Mothers were interviewed with the Structured Clinical Interview for DSM-IV Disorders at an average infant age of *M* = 4.1 months and filled out a questionnaire regarding emotional stress during pregnancy. Further, they were videotaped during the Face-to-Face-Still-Face paradigm (FFSF), a widely used mild socio-emotional stressor for infants. To determine infant stress-reactivity, infant salivary cortisol was collected before, immediately after and 20 min after the FFSF. Missing values were estimated by multiple imputations. At the age of *M* = 5.3 years, mother-child-interaction was re-assessed in a follow-up sample of *n* = 30 dyads via a free-play situation. Moreover, dimensional measures for anxiety were assessed. Mothers in the clinical group reported significantly higher stress scores than the control group. Infant stress reactivity in the early postpartum period and maternal anxiety symptoms at the 5-year follow-up assessment were significantly associated with dyadic interaction quality at pre-school age. Even though maternal stress during pregnancy did not directly predict mother–child interaction quality at pre-school age, it was significantly correlated with infant cortisol reactivity during postpartum period. Nevertheless, caution should be taken when interpreting the results considering the small sample size.

## Introduction

### The Influence of Maternal Stress During Pregnancy on Infant and Child Development

A growing body of research indicates that maternal stress during pregnancy exerts strong influence on the development of the unborn (Van den Bergh et al., 2017). Recent studies underline the long-term influence on a variety of developmental domains in the offspring, such as metabolic functioning, cognitive and emotional development (for review see Beijers et al., 2014). To date, however, the mechanisms through which prenatal maternal stress may affect the unborn are not yet entirely clarified (Hocher, 2014). Among others, prenatal environmental influences, known as fetal programming (Seckl, 2004), genetic factors (Hannigan et al., 2018) as well as postpartum environmental factors (Graignic-Philippea et al., 2014; Mughal et al., 2018) are discussed.

To date, prenatal maternal stress is defined very broadly, including psychological distress such as anxiety or depressive symptoms and life events, e.g., trauma, loss, or natural disasters. In this study we focused on emotional stress during pregnancy. This was assessed retrospectively with a questionnaire in the early postpartum period, including items regarding maternal experience of anxiety, sadness, joy, stress, and general tension (Mohler et al., 2006).

Maternal anxiety disorders in the perinatal period are the most common psychiatric disorders with prevalence rates of 11 to17% (Reck et al., 2008; Fairbrother et al., 2016) and are closely linked with alterations in the human stress systems (Bartlett et al., 2017). The hypothalamic-pituitary- adrenocortical axis (HPA axis) is one major regulating system to cope with stress on hormonal level. Its end product cortisol is intensively discussed as an underlying mechanism accounting for the association between maternal stress/anxiety during pregnancy and infant and child development. Research indicates that elevated maternal cortisol levels in response to stress may affect the offspring’s HPA axis functioning. Consequences might be increased cortisol levels, increased cortisol reactivity (Luecken et al., 2013; Zijlmans et al., 2015) and, on the long run, an increased risk for developmental problems in the offspring. Evidence, however, is still inconsistent as there are recent studies pointing in the opposite direction (O’Connor et al., 2013; Nazzari et al., 2019). The clinical sample in our study consisted of mothers with anxiety disorders, the vast majority (92,8%) with a prepartum onset and ongoing diagnoses in the postpartum period. Therefore, we assume substantial continuity in maternal stress from pregnancy to the first months postpartum suggesting that prenatal stress is strongly associated with maternal postnatal experiences.

Even though it is difficult to disentangle the influence of prenatal and postnatal maternal stress on infant and child development, current studies try to differentiate between timing effects. Prenatally, maternal emotional stress seems to affect cognitive development. In a study of Lin et al. (2017) prenatal emotional stress induced cognitive deficits independent of postnatal stress, even though, maternal pre- and postnatal emotional stress levels were moderately correlated. Regarding maternal prenatal anxiety, recent studies reported that maternal anxiety during pregnancy is associated with socio-emotional problems (Madigan et al., 2018) and a more difficult temperament in the offspring (e.g., high negative affectivity and poor attentional regulation) (Baibazarova et al., 2013; McMahon et al., 2013). A study of Chong et al. (2016) showed that this prenatal effect was independent of maternal anxiety postpartum. Both, maternal prenatal anxiety and emotional stress are associated with higher reaction intensity in children (Lin et al., 2017), higher negative emotionality (Pluess et al., 2010), significant higher rates of behavioral problems and lower prosocial behavior in 5 year-old children independent of concurrent maternal mood (Loomans et al., 2011) and predicted poorer working memory at 8 years of age (Pearson et al., 2016).

A growing body of studies find significant links between prenatal stress and/or anxiety and child development up to adolescence (for review see, Loomans et al., 2011; Graignic-Philippea et al., 2014) Furthermore, maternal anxiety disorders are discussed to increase the offspring’s risk for the development of anxiety disorders (Hettema et al., 2001; Low et al., 2012; Lebowitz et al., 2016). However, caution is required in the assumption of causality as all studies are of observational character.

To the best of the author’s knowledge, there is only one study investigating prenatal maternal stress, as reflected in higher depressive and anxious symptoms, on mother–child interaction at pre-school age. In this study by Endendijk et al. (2017) prenatal emotional symptoms were not related to the quality of the mother–child interactive behavior at the age of 23–60 months. As far as known, there are no studies taking postnatal anxiety disorders according to DSM-IV and infants stress reactivity into account.

### The Role of Maternal Sensitive Interaction Behavior for Infant Stress Regulation

To pacify stressful experiences and to regulate their own affects, infants only have a limited repertoire of self-regulatory behaviors during the first months of life, such as hand-to-mouth-movements and non-nutritive sucking. Therefore, infants depend on the co-regulation of their caregivers, playing an important role in the development of stress regulation in infants (Provenzi et al., 2018). Empirical findings highlight the importance of specific patterns of mother–child interaction for infants’ affect regulation. For dyadic co-regulation, sensitive reactions of the caregivers are of special importance for the development of affect regulation in the infant as well as for behavioral and physiological reactions (Haley and Stansbury, 2003; Conradt and Ablow, 2010). Sensitive and responsive parents are described as paying attention toward the infant’s signals and reacting promptly and appropriate to them. If the caregiver cannot respond adequately to the child’s emotions and interpersonal regulation fails, infants engage in self-directed stress regulation and develop lower tolerance to negative affect and lower stress regulation competencies (Gianino and Tronick, 1988; Tronick, 1989; Müller et al., 2016).

For a better understanding about underlying mechanisms through which maternal stress or anxiety affects child development (Leclere et al., 2014; Pratt et al., 2017), it might be helpful to observe dyadic interactional codes, which address the mother–child dyad as a single unit. On the one hand, we therefore focused on dyadic reciprocity which is described as a mutual exchange in which each interaction partner contributes and the interaction is characterized by collaboration and joint activity (Feldman et al., 2013). On the other hand, special attention was paid to dyadic negative states. The interaction behavior of dyads displaying high dyadic negative states is constricted and poor of emotional expressiveness or enthusiasm. Further, the atmosphere is tense and it seems that mother and child feel uncomfortable with each other (Feldman, 1998).

Overall, maternal sensitivity does not seem to be generally limited in anxiety disorders as studies display a heterogeneous picture. Some studies reported less sensitive maternal interaction behavior in mothers with anxiety disorders compared to healthy controls (Warren et al., 2003; Feldman et al., 2009) while others did not find differences regarding maternal sensitive or intrusive behaviors (Murray et al., 2007, 2012; Weinberg et al., 2008; Kaitz et al., 2010). However, some studies suggested that anxious mothers spoke in a less positive emotional tone (Nicol-Harper et al., 2007), smile less, played or imitated their infants less frequently (Field et al., 2005) and showed more anxious facial expressions (Murray et al., 2007) compared to healthy control dyads. Infants of highly anxious mothers showed less positive affect, more withdrawal, more frequent crying, and increased signs of stress during interaction (Stein et al., 2012). As the quality of interaction is of such great importance for infant stress regulation (Bosquet Enlow et al., 2014), infants of anxious mothers might frequently lack sufficient regulatory scaffolding with possible long term consequences for child socio-emotional development (Martinez-Torteya et al., 2014). This interactive dysregulation might be partly responsible for the increased risk for the development of mental disorders in infants of anxious caregivers (e.g., Mäntymaa et al., 2009).

So far, no study has investigated the links between prenatal stress and the quality of mother–child interaction at pre-school age, taking postpartum anxiety disorders into account. As one of the first studies we wanted to focus on maternal emotional stress during pregnancy in a sample of postpartum anxious mothers and its association’s with mother-child-interaction at pre-school age taking infant stress reactivity during infancy into account. We assumed that emotional stress during pregnancy would differ significantly in both groups, with mothers in the clinical group reporting higher stress scores. We also expected that prenatal stress would be significantly associated with infant stress reactivity in early infancy. Furthermore, we hypothesized that emotional stress during pregnancy as well as infant cortisol reactivity and maternal anxiety would predict mother–child interaction quality at the 5-year follow-up assessment.

## Materials and Methods

### Participants

This sample (*N* = 63 at study entry) is part of a larger longitudinal study (Reck et al., 2013, 2018a,b; Tietz et al., 2014). Participants were recruited between June 2006 and October 2010. They were reached by distribution of flyers (e.g., to gynecologists) as well as newspaper advertisements. Furthermore, the study team responded to public birth announcements and cooperated with the Heidelberg University Women’s Hospital in order to recruit women with anxiety disorders for the clinical group. The clinical group consisted of women diagnosed with at least one of the following anxiety disorders according to DSM-IV: panic disorder with agoraphobia, agoraphobia without history of panic disorder, generalized anxiety disorder, social phobia, obsessive compulsive disorder, post-traumatic stress disorder, and anxiety disorder not otherwise specified (NOS). They were excluded if an acute or former psychosis, a current or former bipolar disorder, current substance abuse, acute suicidal tendency or an acute major depression or dysthymia were diagnosed as primary disorder. Provided the anxiety disorder posed as primary diagnosis, women were not excluded. This was the case for *n* = 4 women (*n* = 1 major depression, *n* = 2 dysthymia, *n* = 1 depressive disorder NOS). Women were only included in the control group if they had no current or antecedent axis I diagnoses according to DSM-IV.

**FIGURE 1:**
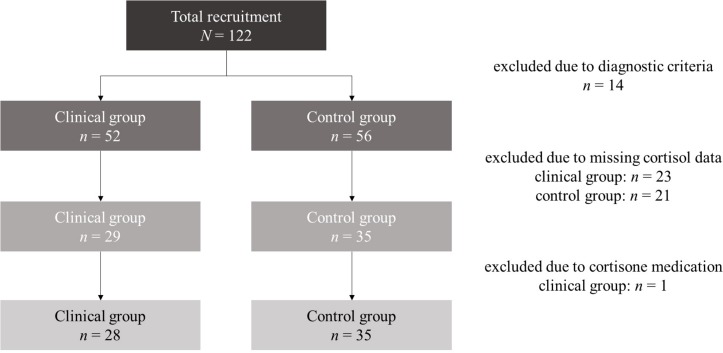
Flow chart of recruitment process and case exclusions.

At first assessment *N* = 122 mothers with their infants were reached. For the present analyses, we excluded *n* = 14 dyads who met diagnostic exclusion criteria. In the remaining subsample (*N* = 108), *N* = 64 mothers agreed upon selection of their infant’s saliva. *n* = 1 infant was excluded due to cortisone medication. None of the infants met the exclusion criteria of prematurity (born before the completion of the 36^th^ week of gestation), small-for-gestational-age or congenital abnormalities. Thus, the final sample consisted of *N* = 63 mother–infant dyads at study entry. *n* = 28 of the mothers were diagnosed with at least one anxiety disorder (clinical group) and *n* = 35 mothers had no clinical disorder (control group). A detailed report of total recruitment and case exclusions is demonstrated in Figure 1.

The distribution of diagnoses in the clinical group was as follows: *n* = 20 mothers had more than one anxiety disorder: *n* = 14 mothers had a panic disorder with or without agoraphobia or an agoraphobia without history of panic disorder. *n* = 14 mothers were diagnosed with a generalized anxiety disorder. *n* = 13 mothers were diagnosed with an obsessive-compulsive disorder. *n* = 9 mothers had a social phobia. *n* = 9 women were diagnosed with a specific phobia. *n* = 1 woman had a post-traumatic stress disorder. *n* = 2 mothers had an anxiety disorder NOS. The vast majority of the clinical sample (*n* = 26, 92,8%) was diagnosed with a prepartum onset of their anxiety disorder(s).

At the 5-year follow-up, only a sub-sample of *N* = 32 mother–infant dyads could be reached (*n* = 19 control group; *n* = 13 clinical group). This high dropout rate was primary due to life changes in the recruited families (change of address and contact data) and changes of interests (rejection of or non-response to study invitation) within the assessment gap of 5 years. *n* = 6 still suffered from at least one current anxiety disorder and further *n* = 3 from at least one partially remitted anxiety disorder. Of these, *n* = 1 mother had four and *n* = 1 mother had three anxiety disorders. Overall, there was *n* = 1 mother with a current agoraphobia without history of panic disorder and *n* = 1 mother with a fully remitted panic disorder. *n* = 2 women were diagnosed with a current generalized anxiety disorder (*n* = 1 partially remitted); *n* = 6 women suffered from an obsessive-compulsive disorder (*n* = 4 partially remitted); *n* = 1 woman had a social phobia; *n* = 2 mothers were diagnosed with a specific phobia; and *n* = 1 woman was diagnosed with a partially remitted anxiety disorder NOS. Maternal and infant characteristics at both assessment points and stratified for both groups are presented in Table 1. Of *n* = 2 mothers the interactive situation was missing at the 5-year follow-up. Consequently, only *N* = 30 dyads were analyzed for dyadic interactive abilities.

**TABLE 1:** Demographics and tests on comparability of subgroups.

	**General**	**Control**	**Anxiety**	***t (p)***
Infant age at study entry (months)^a^ *M* (*SD*)	4.1 (1.4)	4.0 (1.4)	4.3 (1.5)	−0.92 (0.36)
Gestation age (weeks)^b^ *M (SD)*	39.5 (1.5)	39.7 (1.3)	39.2 (1.6)	1.31 (0.18)
APGAR (average)^c^ *M (SD)*	9.5 (0.6)	9.5 (0.6)	9.5 (0.6)	0.07 (0.94)
Child age at 5-year-follow-up (years)^d^ *M* (*SD*)	5.3 (0.4)	5.3 (0.4)	5.5 (0.5)	−1.25 (0.22)

**Infant gender (frequencies)**	**General**	**Control**	**Anxiety**	**χ^2^ (*p*)**

Female	42	24	18	0.13^e^ (0.79)
Male	21	11	10	
	**General**	**Control**	**Anxiety**	***t (p)***
Maternal age at study entry (years) ^f^ *M* (*SD*)	32.4 (5.6)	33.0 (5.5)	31.8 (5.6)	0.89 (0.38)
Maternal age at 5-year-follow-up (years) ^g^ *M* (*SD*)	39.8 (5.6)	40.2 (5.3)	39.3 (6.2)	0.46 (0.65)

**Maternal education (frequencies)**	**General**	**Control**	**Anxiety**	***U (p)***

University degree	34	19	15	463.0 (0.70)
University entrance qualification	12	8	4	
High secondary qualification	14	7	7	
Low secondary qualification	3	1	2	

**Marital status (frequencies)**	**General**	**Control**	**Anxiety**	**χ^2^ (*p*)**

Married	42	26	16	2.15^h^ (0.23)
Not married	15	6	9	

*^*a*^Min = 2.5; max = 7.8; ^*b*^min = 36.3; max = 41.9; ^*c*^min = 7.0; max = 10.0; ^*d*^min = 5.01; max = 6.06; ^*e*^0 cells has expected count less than 5, minimum expected count is 9.33; ^*f*^min = 22.0; max = 45.0; ^*g*^min = 27; max = 50; ^*h*^0 cells have expected count less than 5, minimum expected count is 6.58.*

### Procedures

Assessments took place at the Heidelberg University Hospital, Germany. The study was approved by the independent ethics committee of the Heidelberg University Medical Faculty. According to the Declaration of Helsinki, written informed consent was obtained from all mothers both for their own participation as well as for the participation of their infant prior to the first assessment and after a full explanation of all study procedures. At first assessment, the Face-to-Face Still-Face paradigm (FFSF; Tronick et al., 1978) was videotaped. The FFSF paradigm consists of three episodes, 2 min each (for a detailed description see Tronick et al., 1978). Afterward, the Structured Clinical Interview-I for DSM-IV Disorders (SCID-I, Wittchen et al., 1997) was carried out. Questionnaires were filled out at home and sent back via mail. Five years postpartum, mother–child dyads were re-invited to the lab. At this follow-up assessment, mothers and their children engaged in a 20-min free-play situation which was video-recorded. Following, mothers again were interviewed via the SCID-I and filled out questionnaires at home.

### Infant Cortisol Reactivity

According to recent literature at the time of study planning (for review see Gunnar et al., 2009) infant salivary cortisol was collected immediately before (C_1_), immediately after (C_2_) and 20 min after the FFSF paradigm (C_3_). Even though the circadian rhythm of the HPA-axis is not fully developed during the first months postpartum (de Weerth et al., 2003; Bright et al., 2012) salivary cortisol can be seen as a valid marker for infant stress reactivity in early infancy (Jansen et al., 2010). Saliva samples were stored at −20°C until required for analysis according to standard procedures (Schwartz et al., 1998) with a detection limit of the used assay between 0.1 and 15.0 ng/ml and intra-assay variances of 5.95% Vol. for 2.6 μg/100 ml, 1.59% Vol. for 17 μg/100 ml and 4.62% for 26.6 μg/100 ml). In order to control for effects of circadian rhythm on cortisol reactivity, the assessments were scheduled between 10.00 and 11.00 AM (*M* = 11.0 AM, *SD* = 1.8 h). However, *n* = 14 infants (22.2% of study sample) were assessed after 11 AM (*M* = 13.8 AM, *SD* = 1.2 h) due to postponed study appointments. Thus, we considered sampling daytime as a confounder. Furthermore, cortisol reactivity is correlated with prior napping and feeding. Consequently, additionally to the instruction of keeping the infants rested and fed as usually, time to and length of prior feeding and sleeping periods were considered as additional confounders. Salivary cortisol levels were interpreted on the basis of the area under the curve with respect to increase (AUC_I_) which allows a sensitive measure of physiological changes over time (Pruessner et al., 2003).

For two infants the C_1_ and the C_2_ as well as for *n* = 18 infants the C_3_ value was missing due to low amounts of saliva or interruption of assessment by feeding or napping. Mean values in all measurements (C_1_: *M* = 1.34 ng/ml, *SD* = 1.35 ng/ml, min = 0.10 ng/ml, max = 7.10 ng/ml; C_2_: *M* = 1.31 ng/ml, *SD* = 1.28 ng/ml, min = 0.10 ng/ml, max = 6.50 ng/ml; C_3_: *M* = 1.07 ng/ml, *SD* = 0.98 ng/ml, min = 0.10 ng/ml, max = 3.90 ng/ml) were in range of normative parameters (Tollenaar et al., 2010). Mean of the AUC_I_ was negative (*M* = −4.65 ng/ml × min, *SD* = 17.08 ng/ml × min, min = −46.00 ng/ml × min, max = 39.20 ng/ml × min), indicating that cortisol levels declined from the first (C_1_) to the last (C_3_) assessment. In the group of infants with three valid cortisol measurements (*n* = 42) there were *n* = 13 responders (31%); responders were defined as infants whose AUC_I_ was one SE (2.63 ng/ml × min) above zero. Low to medium responder rates and consequently declining cortisol means are often found in infant and child samples (Gunnar et al., 2009; Jansen et al., 2010). However, all valid infant AUC_I_ values were considered for the analyses. No outlying values (defined as values deviating more than three interquartile ranges from the median) were identified for the AUC_I_ measure. Furthermore, there were no significant correlations (all *p* > 0.15) with potential confounder variables (infant and maternal age, infant gender, marital status, financial concerns, gestational age, PDA, breastfeeding, number of infants, APGAR values, daytime of assessment, time passed since and duration of prior meal or sleeping periods, frequency and duration of daytime naps and nighttime awakes, sleeping arrangement and childcare). Consequently, we disregarded these factors as confounders for the main and additional analyses.

### Maternal Anxiety Disorders

The German version of the Structured Clinical Interview for DSM-IV Axis I Disorders (SCID-I, Wittchen et al., 1997) is semi-structured and a widely used interview for the diagnosis of selected Axis I disorders. It was used by trained and experienced clinical psychologists for the assessment of maternal postpartum anxiety disorders. According to DSM-IV, we included participants with the following diagnoses: generalized anxiety disorder, panic disorder with and without agoraphobia, agoraphobia without history of panic disorder, specific phobias, social phobia, obsessive-compulsive disorder, post-traumatic stress disorder, and the anxiety disorder NOS.

### Maternal Anxiety Symptoms: Agoraphobic Cognitions Questionnaire (ACQ), Body Sensations Questionnaire (BSQ) and the Mobility Inventory (MI: MIA/MIB)

Core symptoms of anxiety disorders at the 1^st^ assessment and the 5-year follow-up were assessed with the German version of the Agoraphobic Cognitions Questionnaire (ACQ), the Body Sensations Questionnaire (BSQ) and the Mobility Inventory (MI) (Ehlers et al., 2001). The three questionnaires combine cognitions, physical symptoms and avoidance behavior, all core symptoms of anxiety disorders. The ACQ consists of 14 questions about the frequency of typical anxiety cognitions with scales ranging from 1 (“thought never occurs”) to 5 (“thought always occurs when I am nervous“). The BSQ consists of 17 items and measures the extent of fear of anxiety related physical symptoms from 1 (“not frightened or worried by this sensation”) to 5 (“extremely frightened by this sensation”). The MI consists of 27 items describing predominant agoraphobic situations and the avoidance of these situations. Moreover, it also indicates the severity of the anxious symptomatology. The MI is subdivided into the Mobility Inventory Alone (MIA) and the Mobility Inventory Backened (MIB; i.e., accompanied by a person trusted). In our sample, internal consistency ranged from Cronbach’s α = 0.88 (ACQ at both assessments) to 0.95 (MIB at both assessments); MIA = 0.95 at 1^st^ assessment and 0.94 at 5-year follow-up; BSQ = 0.92 at both assessments. Thus, the internal consistency was comparable to the ones reported by the authors and can be evaluated as good to excellent.

The mean scores for the anxiety questionnaires at the 1^st^ assessment were as follows: ACQ: *M* = 1.33 (*SD* = 0.38, min = 1.00, max = 2.50), MIA: *M* = 1.31 (*SD* = 0.53, min = 1.00, max = 3.56), MIB: *M* = 1.11 (*SD* = 0.22, min = 1.00, max = 2.12), BSQ: *M* = 1.69 (*SD* = 0.59, min = 1.00, max = 3.53). Anxiety questionnaire data was missing for *n* = 3 women in the 5-year follow-up sample, thus the sample size for analyses controlling for current anxiety symptoms was reduced. The mean scores for the anxiety questionnaires at the 5-year follow-up were as follows: ACQ: *M* = 1.31 (*SD* = 0.44, min = 1.00, max = 3.07), MIA: *M* = 1.30 (*SD* = 0.46, min = 1.00, max = 2.96), MIB: *M* = 1.13 (*SD* = 0.30, min = 1.00, max = 2.42), BSQ: *M* = 1.68 (*SD* = 0.63, min = 1.00, max = 3.41). In this study, we used a composite score averaged over the four scales for each assessment to encounter symptom heterogeneity between the different anxiety disorders. This composite score reached an excellent internal consistency (α = 0.99 at 1^st^ assessment; α = 0.93 at 5-year follow-up) and ranged from min = 1.02 to max = 2.51 (*M* = 1.33, *SD* = 0.38) at the 5-year follow-up respectively from min = 1.00 to max = 2.36 (*M* = 1.33, *SD* = 0.33) at the 1^st^ assessment.

### Prenatal Emotional Stress Index

The Prenatal Emotional Stress Index (PESI) is a self-report questionnaire assessing emotional stress during pregnancy (Mohler et al., 2006). It was applied at the first measurement time to assess maternal emotional stress during pregnancy. retrospectively. The questionnaire consists of 11 items per pregnancy trimester (overall 33 items) measuring anxiety, sadness, joy, stress, and tension via visual analogous scales ranging from 0 to 100%. The scale values are the mean item responses per trimester. Cronbach’s α was α = 0.91 for the first, α = 0.92 for the second and α = 0.93 for the third trimester, indexing excellent internal consistency. Average scores were *M* = 31.92 (*SD* = 24.60, min = 0,00, max = 92.27) for the first, *M* = 30.41 (*SD* = 22.59, min = 0,00, max = 89.09) for the second and *M* = 31.63 (*SD* = 22.93, min = 0,00, max = 91.27) for the third trimester. The intercorrelations between pregnancy trimesters were as follows: the first trimester significantly correlated with the second (*r* = 0.76, *p* < 0.01) and third trimester (*r* = 0.70, *p* < 0.01) as well as the second trimester correlated significantly with the third trimester (*r* = 0.82, *p* < 0.01). A composite score averaged over the three trimesters was used for the analyses This composite score reached an excellent internal consistency (α = 0.96) and ranged from min = 2.42 to max = 86.97 (*M* = 31.34, *SD* = 21.41).

### Mother–Child Interaction

At the 5-year follow-up mother–child interaction during a free-play situation was coded by a trained and reliable coder (inter-rater-consistency = 87.6%) using the 4^th^ revision of the macro-analytical tool “Coding Interactive Behavior” (CIB) (Feldman, 1998). The coder was blind to the study hypotheses and maternal psychiatric status.

The CIB is a valid, reliable and widely used (Feldman, 1998) global rating system for interaction sequences between two or more partners. The basic coding scheme consists of 48 scales (22 for adults, 16 for children and 5 dyadic scales), which are scored from 1 to 5 in steps of 0.5 (1 = minimal level of specific behavior/attitude; 5 = maximal level of specific behavior/attitude). The scales assess the macro-analytical nature and flow of the interaction (e.g., reciprocity and adaption) as well as the involvement and interactive style of each partner (e.g., specific behaviors and affective/attentive states). The different scales can be analyzed separately or averaged to 8 composites (3 for both caregiver and child and 2 dyadic composites).

In this study, we focused on both dyadic composites, i.e., “reciprocity” (averaged from the scales “dyadic reciprocity,” “adaption-regulation,” and “fluency”) and “dyadic negative states” (averaged from the scales “constriction” and “tension”). The means were *M* = 3.67 (*SD* = 0.71, min = 2.17, max = 4.83) for reciprocity and *M* = 1.70 (*SD* = 0.82, min = 1.00, max = 3.50) for dyadic negative states.

### Statistical Analyses

For all analyses we used the *Statistical Package for Social Sciences* (IBM^TM^ SPSS^®^ v. 24.0.0.0). G-Power v. 3.1.9.2 (Faul et al., 2007, 2009) was used for power-estimations for the confirmative analysis.

Before carrying out the main analyses, we tested if the list-wise case-exclusions (see section “Participants”) were valid for our sample and analyses by the use of Little’s MCAR-test (Little, 1988). If the MCAR-test is non-significant it is unlikely that excluded cases and the final sample differ regarding considered variables. We considered socio-demographic data (e.g., age), birth data (e.g., gestational age), self-report data (e.g. PESI), interactive variables (ICEP-R, Reck et al., 2009), cortisol data (and potential confounders) and interaction data (CIB) at the 5-year follow-up for this procedure. Moreover, child and gestational age, APGAR values, child gender, maternal age and education as well as marital status were checked for differences between the included and excluded cases as well as between the control and the clinical group via *t*-tests, *U*-tests and χ^2^-tests to ensure comparability.

For the main analysis, Mann–Whitney *U*-tests were carried out to compare the study groups regarding emotional stress, infant cortisol reactivity as well as CIB scores as the distributions of questionnaire data, cortisol values and reactivity as well as dyadic negative states significantly deviated from normal distribution (*p* < 0.01 in Kolmogorov–Smirnov and Shapiro–Wilk test). Especially for small samples and unequally sized groups, general linear modeling may not be sufficiently robust against violations to mathematical assumptions (e.g., normal distribution). Thus it may lead to progressive statistical testing (Bortz and Schuster, 2010). The intercorrelation between variables indexing for symptom severity, i.e., emotional stress (PESI total score) and anxiety symptoms (at both assessments) were explored via Pearson correlations. Furthermore, the association between emotional stress and infant cortisol reactivity was analyzed via generalized linear modeling (with maximum likelihood estimation and stepwise backward procedure) to control for and to evaluate the independent contribution of study variables (emotional stress and anxiety symptoms). Variables were used uncentered. Consequently, the *B*-weights of the regression models were not standardized. To estimate effect sizes, we computed Cohen’s d for group comparisons and *w*^2^ ($=\frac{\chi^{2}}{N}$) for regression coefficients. *d* = 0.2 respectively *w*^2^ = 0.01 are interpreted as small, *w*^2^ = 0.09 respectively *d* = 0.5 as medium-sized and *w*^2^ = 0.25 respectively *d* = 0.8 as large effects (Cohen, 1977).

Effects of group, measurement time and their interaction term on infant cortisol measures were tested by a two-way ANOVA for repeated measures with the between-subject factor “group” and the within-subject factor “measurement time” to account for differences in the infants’ cortisol trajectories between the groups and for intraindividual variance. Since mathematically premises are violated (see above) the results of this ANOVA are of purely descriptive quality. Mauchly’s procedure was used to test for violation of the assumption of sphericity. This assumption was violated for infant cortisol values (pooled data: *p* < 0.01; ε = 0.71; original data: *p* < 0.01; ε = 0.77). Consequently, repeated measures dfs were Huynh–Feldt corrected.

For the further additional analyses, generalized linear modeling (with maximum likelihood estimation and stepwise backward procedure) was used to control for and to evaluate the independent contribution of study variables (emotional stress, cortisol reactivity, and anxiety symptoms) in explaining the CIB scores.

We used a two-tailed critical α-error of α = 0.05. The valid sample size varies as a function of missing values in the original data and the 5-year-follow-up.

## Results

### Preliminary Data Analyses

The MCAR-test was non-significant (χ^2^ = 1,071.80, *df* = 1,168, *p* = 0.98), indicating that the list-wise case-exclusions were valid for our sample and that this sub-population was representative for the total sample. Additionally, tests on comparability between the excluded and included cases revealed no systematic differences (all *p* > 0.13). Moreover, no differences were found between the groups regarding sociodemographic or birth-related data (see Table 1).

We had complete cortisol data (i.e., to all three measurement points C_1_, C_2_, and C_3_) for *n* = 42 infants (66.7% of study sample). However, the infants of the remaining sample (*n* = 21) had at least one valid cortisol value. Furthermore, *n* = 8 PESI-scores (12.7% of study sample) for each trimester as well as anxiety questionnaire scores at the first assessment (ACQ: *n* = 8, MIA: *n* = 15, MIB: *n* = 13, BSQ: *n* = 8; 12.7–23.8% of study sample) were missing. We estimated the missing values for these dyads using multiple imputations (Rubin, 1987). For these estimations, all variables analyzed in this study were used as predictors according to standard procedures (Enders, 2010). We estimated missing values for *N* = 20 data sets (automatic imputation method, linear regression model, maximum 10 iterations). Estimated cortisol values were restricted to the limit of detection of the cortisol assay (0.1–15.0 ng/ml), PESI and anxiety questionnaire scores were restricted to the maximum score range (PESI: 0–100%, anxiety questionnaires: 1–4). Table 2 contains the means and standard deviations of the pooled imputed values and the pooled sample after multiple imputations. There were no systematic variations of estimated values in the iteration process. Variation occurred within the scope of random variations. The AUC_I_, the PESI and the anxiety symptoms composite were then additionally computed for every case including cases with imputed values in every imputed data set.

**TABLE 2:** Pooled imputation result (averaged over 20 data sets) of missing data.

	**Imputed values**				**Data after imputation (*N* = 63)**			
**Assessment**	***M***	***SD***	**Min**	**Max**	***M***	***SD***	**Min**	**Max**
C_1_ (in ng/ml) (*n* = 2 imputed values)	1.49	0.95	0.81	2.16	1.34	1.34	0.10	7.10
C_2_ (in ng/ml) (*n* = 2 imputed values)	1.15	0.60	0.73	1.58	1.31	1.27	0.10	6.50
C_3_ (in ng/ml) (*n* = 18 imputed values)	1.19	0.84	0.10	2.75	1.10	0.94	0.10	3.90
PESI 1st trimester (in%) (*n* = 8 imputed values)	31.53	4.84	25.05	39.41	31.87	23.03	0.00	92.27
PESI 2nd trimester (in%) (*n* = 8 imputed values)	30.36	4.84	23.06	37.36	30.40	21.16	0.00	89.09
PESI 3rd trimester (in%) (*n* = 8 imputed values)	32.17	5.08	24.21	39.04	31.70	21.49	0.00	91.27
PESI total score	30.92	4.64	24.23	37.59	31.29	20.05	2.42	86.97
ACQ (*n* = 8 imputed values)	1.42	0.43	1.00	2.14	1.34	0.37	1.00	2.50
MIA (*n* = 15 imputed values)	1.48	0.53	1.00	2.65	1.36	0.54	1.00	3.56
MIB (*n* = 13 imputed values)	1.29	0.32	1.00	1.93	1.15	0.26	1.00	2.12
BSQ (*n* = 8 imputed values)	1.79	0.66	1.05	2.85	1.71	0.60	1.00	3.53
Anxiety symptoms (composite)	1.43	0.25	1.07	1.98	1.35	0.31	1.00	2.36

Since the missing interaction data of the 5-year follow-up in *n* = 33 dyads (52.4% of study sample) was not due to just missing values but real study dropouts over an extended period and since the percentage of missing data exceeded 50%, we refrained from estimating missing values for the 5-year follow-up. All analyses were carried out for (1) the original data set and (2) in each of the 20 imputed data sets. The results of (2) were pooled. Consequently, two results are reported: the pooled results over the imputed data set and the results of the original data set.

### Main Analyses

Table 3 summarizes the Mann–Whitney *U*-tests on comparisons between the clinical and the control group. Only emotional stress during pregnancy (PESI total score) differed between the groups in the pooled (*p* < 0.01) and the original data set (*p* < 0.01). The clinical group reported higher scores of emotional stress compared to controls (Cohen’s *d* pooled data: *d* = 1.75; original data: *d* = 2.19). There were no group differences between the clinical and the control group regarding infant cortisol reactivity (AUC_I_; pooled data: *p* = 0.19; original data: *p* = 0.66) or CIB scores (dyadic negative states: *p* = 0.21; dyadic reciprocity: *p* = 0.62).

**TABLE 3:** Mann–Whitney *U*-tests on differences between the study groups regarding emotional stress during pregnancy (PESI total score), infant cortisol reactivity (AUC_I_), and dyadic interaction (CIB) at 5 years follow-up.

		**Control Group**		**Clinical Group**		**Test statistic**	
**Outcome**		***M***	***SE***	***M***	***SE***	***U***	***p***
Emotional stress (PESI total score)	Original data (*n* = 55)	19.37	1.77	52.29	4.37	40.00	<0.01
	Pooled data (*n* = 63)^a^	19.37	1.77	46.18	3.66	75.86	<0.01
Infant cortisol reactivity (AUC_I_)	Original data (*n* = 42)	–5.56	2.89	–3.18	5.19	191.00	0.66
	Pooled data (*n* = 63)^a^	–5.38	3.22	0.18	3.88	374.52	0.19
Dyadic negative states (CIB)	Original data (*n* = 30)	1.54	0.18	1.94	0.26	80.50	0.21
Dyadic reciprocity (CIB)	Original data (*n* = 30)	3.75	0.14	3.54	0.26	96.50	0.62

*^*a*^For pooled analyses: averaged over original and imputed data sets.*

The intercorrelation between emotional stress and anxiety symptoms at the 1^st^ assessment (pooled data: *r* = 0.631, *p* < 0.01; original data: *r* = 0.709, *p* < 0.01) and the 5-year follow up (pooled data: *r* = 0.621, *p* < 0.01; original data: *r* = 0.625, *p* < 0.01) was positive and significant. The intercorrelation between anxiety symptoms at the 1^st^ assessment and the 5-year follow-up was positive and significant in the pooled (*r* = 0.636, *p* < 0.01) and the original data set (*r* = 0.709, *p* < 0.01).

To evaluate the unique and independent association between emotional stress during pregnancy (PESI total score) and infant cortisol reactivity (AUC_I_), we used a generalized linear backward regression controlling for anxiety symptoms at the 1^st^ assessment. As maternal disorder was not revealed as meaningful for infant cortisol reactivity, it was neglected in this analysis. In the first step, anxiety symptoms at the 1^st^ assessment were excluded as non-significant for infant cortisol reactivity (pooled data: *B* = −0.920; *p* = 0.928; original data: *B* = −6.479; *p* = 0.384). The second and final step (Table 4, Model 1) contained only emotional stress as significant predictor for infant cortisol reactivity: Emotional stress was significantly and positively associated with infant cortisol reactivity (pooled data: *p* = 0.036, *w*^2^ = 0.125; original data: *p* = 0.034, *w*^2^ = 0.084).

**TABLE 4:** Generalized linear regression models on infant cortisol reactivity (AUC_I_) and dyadic negative states and reciprocity (CIB).

**Model**		**Parameter**	***B***	***SE***	**Lower *CI* bound (95%)**	**Upper *CI* bound (95%)**	**Wald χ^2 a^**	***p***
Model 1: infant cortisol reactivity (AUC_I_) predicted by emotional stress (PESI total score)	original data (*n* = 36)^b^	PESI total score	0.372	0.175	0.029	0.716	4.512	0.034
		Intercept	–12.350	5.373	–22.881	–1.818	5.282	0.022
		Scale	190.893^*c*^	44.994	120.270	302.984	/	/
	pooled data (*n* = 63)^d^	PESI total score	0.235	0.112	0.015	0.456	5.303	0.036
		Intercept	–10.272	4.459	–19.019	–1.525	6.323	0.021
		Scale	329.473	74.212	182.782	476.163	/	/
Model 2: dyadic negative states (CIB) predicted by anxiety symptoms at the 5-year follow-up and infant cortisol reactivity (AUC_I_)	original data (*n* = 17)^e^	Anxiety symptoms (5-year follow-up)	1.202	0.431	0.356	2.047	7.764	0.005
		AUC_I_	0.016	0.004	0.009	0.023	20.499	<0.001
		Intercept	0.193	0.591	–0.966	1.352	0.106	0.745
		Scale	0.474^*c*^	0.162	0.242	0.928	/	/
	Pooled data (*n* = 25)^f^	Anxiety symptoms (5-year follow-up)	1.280	0.404	0.488	2.072	10.072	0.002
		AUC_I_	0.012	0.004	0.005	0.020	13.186	0.002
		Intercept	0.077	0.529	–0.959	1.114	0.042	0.884
		Scale	0.421	0.120	0.186	0.656	/	/
Model 3: dyadic reciprocity (CIB) predicted by anxiety symptoms at the 5-year follow-up and infant cortisol reactivity (AUC_I_)	original data (*n* = 17)^g^	Anxiety symptoms (5-year follow-up)	–1.196	0.336	–1.854	–0.537	12.659	<0.001
		AUC_I_	–0.009	0.004	–0.018	–0.001	4.928	0.026
		Intercept	5.197	0.482	4.253	6.142	116.330	<0.001
		Scale	0.293^*c*^	0.100	0.150	0.574	/	/
	Pooled data (*n* = 25)^h^	Anxiety symptoms (5-year follow-up)	–1.261	0.325	–1.897	–0.625	15.168	<0.001
		AUC_I_	–0.010	0.004	–0.018	–0.002	7.577	0.010
		Intercept	5.269	0.440	4.407	6.131	144.644	<0.001
		Scale	0.288	0.082	0.128	0.448	/	/

*^*a*^For pooled analyses: averaged over original and imputed data sets; ^*b*^Likelihood-ratio χ^2^ = 6.258, *p* = 0.012; ^*c*^Maximum likelihood estimate; ^*d*^Average likelihood-ratio χ^2^ = 4.348, average *p* = 0.056; ^*e*^Likelihood-ratio χ^2^ = 8.980, *p* = 0.011; ^*f*^Average likelihood-ratio χ^2^ = 12.599, average *p* = 0.002; ^*g*^Likelihood-ratio χ^2^ = 10.371, *p* = 0.006; ^*h*^Average likelihood-ratio χ^2^ = 15.037, average *p* = 0.001.*

Multicollinearity for this model was approximated by linear regression and the estimation of the variance inflation factor (VIF). Multicollinearity seems unproblematic, as the VIF was low for both variables and both data (pooled data: average VIF = 1.670; original data: VIF = 2.011) (O’Brien, 2007).

For the Mann–Whitney *U*-tests, large effects (*d* = 0.8) in our pooled sample could be detected by a chance of 1-β = 0.69 (1-β = 0.30 for minimal data set of *n* = 30). Consequently, especially for medium-sized and small effects the power of these comparisons is not sufficient. The power for the final regression model was approximated for the linear multiple regressions with one coefficient. Large effects (*f*^2^ = 0.35) for beta weights in our pooled sample could be detected by a chance of 1-β = 0.99 (1-β = 0.93 for original data). The chance to find medium-sized effects (*f*^2^ = 0.15) in our pooled sample was 1-β = 0.86 (1-β = 0.62 for original data). Small effects (*f*^2^ = 0.02) had a chance of 1-β = 0.20 (1-β = 0.13 for original data) to be detected. Thus, especially small effects could not be sufficiently detected.

### Additional Analyses

#### Repeated Measures Analysis on Infant Cortisol Reactivity

There was neither a significant main effect of measurement time [pooled data: *F*(1.42,85.32) = 1.83, *p* = 0.24; original data: *F*(1.54,61.67) = 3.24, *p* = 0.06] nor of group [pooled data: *F*(1,61) = 2.40, *p* = 0.14; original data: *F*(1,40) = 0.83, *p* = 0.37]. Moreover, the interaction effect between measurement time and group was non-significant [pooled data: *F*(1.42,85.32) = 1.22, *p* = 0.36; original data: *F*(1.54,61.67) = 0.09, *p* = 0.86]. Means and standard deviations are demonstrated in Table 5.

**TABLE 5:** Descriptive statistics on infant cortisol values by group and measurement time.

			***M***	***SD***	***n***
Original data	C_1_ (in ng/ml)	Control group	1.52	1.50	26
		Clinical group	1.15	0.93	16
		Total	1.38	1.31	42
	C_2_ (in ng/ml)	Control group	1.40	1.45	26
		Clinical group	1.12	0.85	16
		Total	1.29	1.25	42
	C_3_ (in ng/ml)	Control group	1.13	0.98	26
		Clinical group	0.86	0.76	16
		Total	1.03	0.90	42
Pooled data^a^	C_1_ (in ng/ml)	Control group	1.59	1.49	35
		Clinical group	1.03	1.06	28
		Total	1.34	1.34	63
	C_2_ (in ng/ml)	Control group	1.50	1.44	35
		Clinical group	1.06	0.97	28
		Total	1.30	1.27	63
	C_3_ (in ng/ml)	Control group	1.18	1.03	35
		Clinical group	0.99	0.81	28
		Total	1.10	0.94	63

*^*a*^For pooled analyses: averaged over original and imputed data sets.*

Although there is a widely accepted practice of log-transforming raw data to achieve normally distributed values (Miller and Plessow, 2013), this technique is not uncritical regarding its aims, success and its effects on data interpretation, and thus have to be used and interpreted with caution (Feng et al., 2014). However, aiming to assure, that the results of the ANOVA were not referable to skewed cortisol raw values, we applied ln-transformation to our original data. The transformed data were checked for outlying values (as defined by more than 1.5 respectively 3 interquartile ranges below the first respectively above the third quartile). There were neither mild nor extreme outliers. However, after transformation data still were significantly skewed [Komogorov–Smirnov test: *p* = 0.02 for ln(C_1_), *p* = 0.02 for ln(C_2_), *p* = 0.06 for ln(C_3_); Shapiro–Wilk: *p* < 0.01 for all three measures]. The results of the ANOVA (Mauchly’s-test: *p* < 0.01; Huynh–Feldt-ε = 0.86) with the transformed values as dependent variable remained unchanged: There is neither an effect of time [*F*(1.73,69.15) = 3.04, *p* = 0.06], of group [*F*(1,40) = 0.04, *p* = 0.84] nor an interaction effect [*F*(1.73,69.15) = 0.07, *p* = 0.91].

The power to detect large (*f* = 0.40) between-subject effects in this ANOVA was high (pooled data: 1-β = 0.97; original data: 1-β = 0.87. Additionally, large (*f* = 0.40) and medium-sized (*f* = 0.25) within-subject and interaction effects were sufficiently detectable (pooled data: 1-β > 0.97, original data: 1-β > 0.90). Only medium-sized (*f* = 0.25) and small (*f* = 0.10) between-subject effects as well as small (*f* = 0.10) within-subject and interaction effects could not be excluded in these analyses (pooled data: 1-β < 0.67, original data: 1-β < 0.49). It can be concluded that for between-subject effects only large effects can be ruled out. For within-subject effects large and medium-sized effects can be ruled out.

#### Dyadic Interaction Quality

As maternal disorder was not revealed as meaningful for dyadic interaction quality, it was neglected in these additional analyses. To evaluate unique and independent relations of emotional stress during pregnancy (PESI total score), infant cortisol reactivity (AUC_I_) and maternal anxiety symptoms at both assessments with dyadic negative states and dyadic reciprocity (CIB), we used stepwise backward regressions (with generalized linear modeling) with these predictors as main effects.

The following variables were stepwise excluded as non-significant for dyadic negative states: Emotional stress (PESI total score; pooled data: *B* = −0.004; *p* = 0.669; original data: *B* = 0.019; *p* = 0.167) and anxiety symptoms at the 1^st^ assessment (pooled data: *B* = 0.632; *p* = 0.304; original data: *B* = 1.046; *p* = 0.112). For dyadic reciprocity the same variables were excluded, however, in reversed order: Anxiety symptoms at the 1^st^ assessment (pooled data: *B* = −0.260; *p* = 0.681; original data: *B* = −0.121 *p* = 0.864) and emotional stress (PESI total score; pooled data: *B* = 0.009; *p* = 0.104; original data: *B* = −0.001; *p* = 0.918). The third and final step (Table 4, Model 2 for dyadic negative states respectively Model 3 for dyadic reciprocity) contained only significant predictors, i.e., infant cortisol reactivity (AUC_I_; negative states: pooled data: *p* = 0.002, *w*^2^ = 0.527; original data: *p* < 0.001, *w*^2^ = 1.206; reciprocity: pooled data: *p* = 0.010, *w*^2^ = 0.303; original data: *p* = 0.026, *w*^2^ = 0.290) and maternal anxiety symptoms at the 5-year follow-up (negative states: pooled data: *p* = 0.002, *w*^2^ = 0.403; original data: *p* = 0.005, *w*^2^ = 0.457; reciprocity: pooled data: *p* < 0.001, *w*^2^ = 0.607; original data: *p* < 0.001, *w*^2^ = 0.745) were both associated to dyadic interaction quality.

As in these analyses generalized linear modeling was used to minimize effects of violations of mathematical assumptions (Feng et al., 2014) and as non-linear transformations (as, e.g., ln-transformation) change the information regarding equality of numerical differences contained in the data (e.g., relevant for computing AUC_I_-indices), we refrained from repeating these analyses with ln-transformed data despite skewed infant cortisol raw and reactivity values.

Multicollinearity for these models was approximated by linear regression and the estimation of the VIF. For emotional stress (PESI total score: pooled data: average VIF = 1.961; original data: VIF = 2.379), anxiety symptoms at the first assessment (pooled data: average VIF = 2.050; original data: VIF = 2.681), at the 5-year follow-up (pooled data: average VIF = 2.100; original data: VIF = 2.183) as well as for infant cortisol reactivity (pooled data: average VIF = 1.158; original data: VIF = 1.225) multicollinearity seems unproblematic, as the VIF was low for all variables and both data (O’Brien, 2007).

The power for the final models was approximated for the linear multiple regressions with two coefficients. Large effects (*f*^2^ = 0.35) for beta weights in our pooled sample could be detected by a chance of 1-β = 0.87 (1-β = 0.65 for original data). The chance to find medium-sized effects (*f*^2^ = 0.15) in our pooled sample was 1-β = 0.53 (1-β = 0.33 for original data). Small effects (*f*^2^ = 0.02) had a chance of 1-β = 0.12 (1-β = 0.09 for original data) to be detected. Thus, medium-sized and small effects could not be sufficiently detected in our 5-year-follow-up sample.

## Discussion

The present study aimed at evaluating possible long-term consequences of emotional stress during pregnancy and postpartum anxiety disorders on mother–child interaction at pre-school age taking infant stress reactivity during infancy into account. First, our results show that mothers with postpartum anxiety disorders report higher levels of emotional stress during pregnancy. This is in line with previous studies indicating higher levels of perinatal emotional stress in women with postpartum anxiety disorder (Britton, 2008).

Secondly, our study revealed no differences in postpartum cortisol reactivity in infants of mothers with postpartum anxiety disorders and infants of the control group. This is a rather surprising result, as studies describe an association between maternal psychopathology in the postpartum period and the cortisol reactivity of their infants (e.g., Brennan et al., 2008). One explanation for this unexpected result could be that our clinical sample consisted of women with various anxiety disorders. So, it remains unclear whether mothers with different anxiety disorders display the same difficulties regarding mother–infant/child interaction. If the impact of anxiety disorders on mother–infant/child interaction vary this could explain the unexpected result.

Moreover, there might be some methodological reasons for this non-expected finding of our study, for example a missing mean increase in cortisol following the FFSF in our sample. This might be due to the fact, that infant salivary cortisol samples were only taken prior to, immediately after and 20 min after the FFSF. Consequently, we may not have had a full coverage of the possible cortisol peak times, though many studies have used similar intervals as demonstrated in the review of Gunnar et al. (2009). Further research studies should lengthen the observation interval to 30 min as suggested by a review of Provenzi et al. (2016). Second, the finding of a decline in the mean value of infant cortisol-reactivity (AUCI) may be surprising given the established stressful nature of the FFSF. Nevertheless, a low to medium rate of infant cortisol responders (31% for our original data) and thus a decrease in cortisol means often is found in infant and child stress research (Gunnar et al., 2009; Jansen et al., 2010). It must be noted that the lack of reactivity does not imply that the measurement of cortisol reactivity in response to psychological stressors is not meaningful. Rather, it has been argued that the individual differences might bring to light factors that account for the individual differences as well as potential risk factors that may adversely affect infant development. Moreover, a dampening of cortisol-responses to stressors in rodents and humans during early development (Gunnar and Donzella, 2002) might play a role for our results. Although the reasons and duration of this dampening period is still unknown, there are many factors affecting stress reactivity. These include genetic influences (Montirosso et al., 2015), temperament differences (van Bakel and Riksen-Walraven, 2004), age related changes (Jansen et al., 2010), individual differences in sensitivity to the nature of the stimuli and contexts (Gunnar et al., 2009), and a sculpting of stress reactivity by interactive history (Gunnar and Quevedo, 2007). Furthermore, a recent meta-analysis (Provenzi et al., 2016) showed that a robust effect on salivary cortisol reactivity was only found for the 5-episode FFSF studies.

Even though we did not find group differences regarding infant stress reactivity, emotional stress during pregnancy was significantly correlated with infant stress reactivity. This finding adds to the heterogeneous picture about the association between maternal prenatal stress and infant cortisol reactivity. While some studies report an associations between prenatal stress and modified infant stress reactivity (e.g., Luecken et al., 2013), a current systematic review only finds limited evidence for this association (Bleker et al., 2018). As a majority of women in our clinical sample already had perinatal anxiety disorders, it is likely that our results arise from a longer exposure to stress of the fetus due to maternal stress during pregnancy and in the postpartum period. In contrast, time-limited exposure to stress is usually characterized by high chronicity and are often highly inter-correlated across gestation (Davis and Sandman, 2010). Further research is needed to disentangle timing and duration influences of maternal stress on infant stress reactivity.

The results of our study suggest that maternal stress during pregnancy influences infant development (Davis and Sandman, 2010; Buss et al., 2012). This result of our study could suggest programming influences of maternal psychobiological stress response on the developing fetus, but as the results yield from correlative analyses, we cannot draw causal conclusion. A variety of alternative factors, such as genetics, autonomous nervous and immune system functioning (Van den Bergh et al., 2017) as well as methylation processes (Weaver et al., 2004; Oberlander et al., 2008) have to be considered in prospective studies.

In our study, we did not find differences between the clinical and the control group with regard to the quality of interaction at pre-school age. This result was against our hypotheses and could be due to the heterogeneity regarding the anxiety disorders in our sample. Nevertheless, this finding is in line with a number of studies also reporting no difference in mother–infant interaction in mothers with anxiety disorders (Murray et al., 2007, 2012; Weinberg et al., 2008; Kaitz et al., 2010). Although, data about maternal interaction behavior in course of postpartum anxiety disorders is heterogeneous, as some studies did report reduced sensitivity in mothers with anxiety disorders (Feldman et al., 2009).

Notwithstanding, our results show an association between the quality of dyadic interaction at pre-school age and infant cortisol reactivity in the postpartum period as well as current maternal anxiety symptoms. Therefore, our results revealed the crucial role of maternal current anxiety symptoms for dyadic reciprocity as well as for dyadic negative states. Dyads with mothers suffering from current anxiety symptoms show lower mutual exchange, collaboration, and joint activity in interaction. Furthermore, these dyads display poor emotional expressiveness, more feelings of discomfort and the atmosphere is tense. In addition, our results support the assumption, that a dysfunction of the infant’s HPA-axis exerts long-lasting influence on further child development (Lin et al., 2017; Madigan et al., 2018) in dyads with ongoing anxious symptomatology. Our results point toward this direction, as infant cortisol reactivity in the postpartum period significantly predicted child socio-emotional development at pre-school age operationalized as dyadic negative states as well as dyadic reciprocity during mother–child interaction. Both interactive patterns, dyadic negative affect and less dyadic reciprocity, can be seen as markers for a lack of emotion regulation skills (Feldman, 2015). Thus, the present study demonstrates the importance of infant’s HPA functioning for long-term healthy development. This is in line with a current study by Neuenschwander et al. (2018), showing that heightened cortisol reactivity is associated with poorer executive functioning in 6-year old children. Furthermore, children’s cortisol levels functioned as a mediator between maternal prenatal depressed and/or anxious symptoms and executive functioning. Apart from this, our results are in line with recent studies reporting that negative developmental pathways in children with a dysregulation of their HPA-axis function even increase, if familial risks factors are evident, such as parental social anxiety (Poole et al., 2018) or psychosocial risk factors, such as low parental education or unemployment (Karlén et al., 2015).

Contrary to our hypothesis emotional stress during pregnancy did not predict mother–child interaction quality at pre-school age. This is in line with the findings of Endendijk et al. (2017) and leads to the assumption that there must be other factors, for example child temperament, that could account for dysfunctional interactive behavior at pre-school age. In our study, maternal emotional stress during pregnancy was significantly correlated with infant cortisol reactivity during postpartum period, indicating that especially stress during pregnancy might influence stress reactivity during infancy. Therefore, this research issue should be addressed in further research, at best with a mediation analysis, regarding the influence of infant stress reactivity as a potential mediator between prenatal stress and interaction quality at pre-school age. Moreover, the bidirectional nature of the association between child cortisol and interaction quality should be addressed, as some studies highlight the importance of maternal interaction behavior for the regulation of child’s HPA-axis (Muller et al., 2015). Furthermore, future research should address the impact of further confounders, such as child temperament and parenting behavior postpartum. Unfortunately, our study sample was too small to run these analyses, wherefore further research with larger study samples are urgently needed.

The study has some limitations. Firstly, besides a rather small sample size and a low power, especially at the 5-year follow-up, mothers with different anxiety disorders are included in our clinical sample. Furthermore, a majority of women suffered from more than one anxiety disorder. The sample size does not allow subgroup analyses for the different anxiety disorders, and therefore it is impossible to draw conclusions about the specific effect of different anxiety disorders. Secondly, our sample is characterized by an overproportion of academic degrees, whereby our data is not representative for the overall population. Thirdly, the maternal stress questionnaire (PESI) retrospectively assesses emotional stress during pregnancy via self-report which may have affected postpartum answering tendencies. Moreover, it would be of great interest to assess saliva cortisol samples during pregnancy, enabling future studies to analyze the impact of maternal stress during pregnancy for child development prospectively. Fourthly, infant salivary cortisol was assessed prior to, immediately after and 20 min after the Still-Face paradigm. Due to few samples or the small time frame it is possible that we missed peak cortisol reactivity times, which may in part account for the negative mean cortisol reactivity. Lastly, the study design was observational, causality assumptions are not appropriate.

## Conclusion

Taken together, our empirical results as well as theoretical assumptions emphasize the importance to further investigate the influence of stress during pregnancy for infant and child development. Our results underline that emotional stress during pregnancy is linked to infant stress reactivity and this in turn influences mother–child interaction up to pre-school age. Regarding maternal stress and its influences on infant and child development, it would be of major importance to disentangle different time-effects as well as different kinds of stressors, such as psychological stress, anxiety, or depressive symptoms and life events (trauma, loss, or natural disasters). Furthermore, potential moderators should be addressed, such as early life experiences, stress coping strategies, social support or maternal face processing, since current studies show the detrimental effect of anxiety (De Carli et al., 2019). With regard to possible long-term consequences for infant and child development early intervention and prevention programs are of vital importance. Recent studies point toward the direction that especially early interventions focusing dyadic reciprocity could improve children’s regulatory capacities (Feldman, 2015). In sum, the foundation of socio-emotional competencies and especially affect and stress regulation capacities are laid early in life. They are primarily learned in the context of parent-infant/child-interaction with possible long-lasting effects regarding stress regulation for future relationships and mental health over the lifespan.

## Ethics Statement

This study was carried out in accordance with the recommendations of the independent ethics committee of the Heidelberg University Medical Faculty with written informed consent from all subjects in accordance with the Declaration of Helsinki.

## Author Contributions

A-LZ and MM contributed to the analysis and interpretation of the data, drafting of the manuscript, and final approval of the final version of the manuscript. NN and BD contributed to the study and manuscript conception, and approval of the final version of the manuscript. CR contributed to the study conception and design, drafting of the manuscript, and final approval of the final version of the manuscript.

## Conflict of Interest

The authors declare that the research was conducted in the absence of any commercial or financial relationships that could be construed as a potential conflict of interest.
